# A re-assessment of gene-tag classification approaches for describing *var* gene expression patterns during human *Plasmodium falciparum *malaria parasite infections

**DOI:** 10.12688/wellcomeopenres.12053.1

**Published:** 2017-09-19

**Authors:** George Githinji, Peter C. Bull

**Affiliations:** 1 Kenya Medical Research Institute (KEMRI)-Wellcome Trust Research Programme, Centre for Geographic Medicine Research-Coast, Kilifi, Kenya; 2 Department of Pathology, University of Cambridge, Cambridge, UK

**Keywords:** Malaria, PfEMP1, var genes

## Abstract

PfEMP1 are variant parasite antigens that are inserted on the surface of *Plasmodium falciparum* infected erythrocytes (IE). Through interactions with various host molecules, PfEMP1 mediate IE sequestration in tissues and play a key role in the pathology of severe malaria. PfEMP1 is encoded by a diverse multi-gene family called *var*. Previous studies have shown that that expression of specific subsets of *var* genes are associated with low levels of host immunity and severe malaria. However, in most clinical studies to date, full-length *var* gene sequences were unavailable and various approaches have been used to make comparisons between *var* gene expression profiles in different parasite isolates using limited information. Several studies have relied on the classification of a 300 – 500 base-pair “DBLα tag” region in the DBLα domain located at the 5’ end of most *var* genes.

We assessed the relationship between various DBLα tag classification methods, and sequence features that are only fully assessable through full-length *var* gene sequences. We compared these different sequence features in full-length *var* gene from six fully sequenced laboratory isolates.

These comparisons show that despite a long history of recombination, ****DBLα sequence tag classification can provide functional information on important features of full-length *var* genes. Notably, a specific subset of DBLα tags previously defined as “group A-like” is associated with CIDRα1 domains proposed to bind to endothelial protein C receptor.

This analysis helps to bring together different sources of data that have been used to assess var gene expression in clinical parasite isolates.

## Introduction

PfEMP1 is an important target of naturally acquired immunity to malaria ( Chan *et al.*, 2012) and plays a central role in malaria pathology through interaction with host endothelial receptors such as ICAM-1 ( Berendt *et al.*, 1989), CD36 ( Barnwell *et al.*, 1989), CR1 ( Rowe *et al.*, 1997) and endothelial protein-C receptor (EPCR) ( Turner *et al.*, 2013). PfEMP1 undergo antigenic variation through epigenetically controlled, mutually exclusive expression of members of a diverse multi-gene family of around 60 *var* genes in every parasite genome ( Gardner *et al.*, 2002).

Various cytoadhesive functions are encoded by specific PfEMP1 domain subsets. PfEMP1 molecules contain a combination of two to nine domains ( Rask *et al.*, 2010; Smith *et al.*, 2000a) organized in a modular architecture comprising an N-terminal segment, Duffy binding-like (DBL), cysteine inter-domain region (CIDR) and acidic terminal segment domains. DBL domains have been classified into 5 broad groups (α, β, ϒ, δ, ε, and ζ ) ( Smith *et al.*, 2000b) and CIDR domains classified into four broad sub-groups (α, β, ϒ and δ) ( Rask *et al.*, 2010; Smith *et al.*, 2000b) based on sequence similarity. ICAM1 binding is encoded by a subset of DBLβ domains ( Brown *et al.*, 2013), CD36 and EPCR by distinct subsets of CIDRα domains ( Hsieh *et al.*, 2016; Lau *et al.*, 2015) and rosetting by a subset of DBLα domains ( Rowe *et al.*, 1997). Understanding the relationships between specific PfEMP1 variants and clinical malaria is not straightforward, since 1) due to recombination between *var* genes on non-homologous chromosomes, the overall architecture of PfEMP1 encoded by different parasites genotypes is extremely diverse and sequences are mosaics of many semi-conserved sequence blocks, and 2) multiple *var* genes are expressed simultaneously within the infecting parasite population. The range of *var* genes expressed at any one time in the infecting parasite population varies according to the antibodies and other *in vivo* selection pressures. 3) Analysis is further complicated by the high diversity of each domain subclass and lack of clear associations between specific adhesion phenotypes and classes of domains.

Based on full-length sequences from seven laboratory isolates, each domain class has been classified through global sequences alignment into further sub-classes ( Rask *et al.*, 2010). For example, the DBLα domain, which has been reclassified into 33 sub-domains (DBLα 0.1 - 0.24, DBLα 1.1 - 1.8 and DBLα2).

Various broad classification methods have been employed to simplify this complex picture in the hope that a limited set of broad functional specializations may exist within *var* that may clarify the disease process. PfEMP1 genes can be classified in relation to their upstream promoter regions ( *ups*). The *ups* classification partitions the sequences into groups A–E based on the sequence similarity of the 500 base-pair 5’ flanking region and the *var* chromosomal location ( Gardner *et al.*, 2002; Vázquez-Macías *et al.*, 2002; Voss *et al.*, 2003; Voss *et al.*, 2000). *Ups* E is associated exclusively with *var2CSA*, which plays a central role in placental malaria ( Lavstsen *et al.*, 2003). UpsA *var* genes expression has been reported in several studies to be associated with severe disease ( Kyriacou *et al.*, 2006; Lavstsen *et al.*, 2012; Rottmann *et al.*, 2006; Warimwe *et al.*, 2009; Warimwe *et al.*, 2012) and rosetting ( Bull *et al.*, 2005a; Rowe *et al.*, 2002; Warimwe *et al.*, 2012). However, an increased transcription of upsB sequences has also been reported to be associated with severe malaria ( Rottmann *et al.*, 2006). UpsC sequences have been shown to be expressed at higher levels in asymptomatic cases ( Falk *et al.*, 2009; Kaestli *et al.*, 2006); however, expression of upsC sequences in severe malaria cases has also been reported ( Kalmbach *et al.*, 2010).

PfEMP1 can be further described in terms of common configurations of different subclasses of domains. These common configurations have been labelled as “domain cassettes” (DCs) ( Rask *et al.*, 2010). Twenty-three *var* DCs have been defined from full-length domain alignments of sequences from seven laboratory parasites. It was initially proposed that DCs may act as functional units. However, clearly defined functions have only been assigned at the level of individual domain sub-classes. Therefore, though common combinations of domains exist, it is unclear whether they represent functional units. For example: 1) specific CIDRα1 domains often found in the context of domain cassette 8 (DC8) and 13 (DC13) have been found to bind to EPCR ( Turner *et al.*, 2013). *Var* genes containing DC8 cassettes from the IT4 line are suggested to bind to human endothelial cells from various organs and notably from the brain endothelial cells ( Avril *et al.*, 2012; Claessens *et al.*, 2012); 2) DBLβ domains found within DC4 genes were reported to adhere to ICAM-1 and may be targets of broadly cross-reactive and adhesion-inhibitory IgG antibodies ( Bengtsson *et al.*, 2013).

Clinical and laboratory studies have reported associations between DCs and disease severity. Using PCR primers designed to selectively amplify sequence features found within DC8 and DC13, expression of these DCs were found to be associated with severe malaria in a study conducted in Tanzania ( Jespersen *et al.*, 2016; Lavstsen *et al.*, 2012), while a proteomic study in Benin linked the expression of DC8 with cerebral malaria ( Bertin *et al.*, 2013).

Several clinical studies have relied on the classification of DBLα tags ( Kirchgatter & Portillo, 2002; Kyriacou *et al.*, 2006; Warimwe *et al.*, 2009). We have previously classified these tags using two different approaches. In the first approach, we classified tags using the number of cysteine residues they contained and the existence of two mutually exclusive motifs MFK and REY ( Bull *et al.*, 2005b; Bull *et al.*, 2007). Our second approach to classification relied on the fact that recombination between *var* genes appears to be non-random ( Kraemer & Smith, 2003; Kraemer *et al.*, 2007). We used network analysis to define sequence groups that tend to share blocks of sequence with each other. We called the most prominent groups block sharing group 1 and block sharing group 2 (BS1 and BS2), respectively. Block sharing group 1 was found enriched in group-A *var* sequences carrying the *upsA* motif ( Bull *et al.*, 2008). Based on sensitivity and specificity comparisons with known full length sequence data we defined sequences with 2 cysteines (CP1-3) that fell in block sharing group 1 as “group A-like” sequences ( Warimwe *et al.*, 2009). Clinical studies on *var* expression have shown that group A-like sequences are associated with severe malaria ( Warimwe *et al.*, 2009; Warimwe *et al.*, 2012), while two other studies obtained similar results by simply partitioning tags to those with and those without two cysteines ( Kirchgatter & Portillo, 2002; Kyriacou *et al.*, 2006). It is currently unclear whether DBLα tags provide information on specific cytoadhesive phenotypes. Furthermore, Lavstsen *et al.*, 2012 have suggested that information on EPCR binding by CIDRα1 within DC8 and DC13 may be unavailable within the DBLα tag due to a recombination hotspot situated between the DBLα tag region and the CIDRα domain.

In an attempt to bring together information from the DBLα tag with information available from the full length *var* gene, we examined associations between full length *var* gene classifications available from a recent study ( Rask *et al.*, 2010) and *var* tag classifications used in previous studies of clinical parasite isolates ( Bull *et al.*, 2005b; Bull *et al.*, 2007; Kirchgatter & Portillo, 2002; Kyriacou *et al.*, 2006; Warimwe *et al.*, 2009).

## Methods

### Data collection and sequence classification

DBLα sequence tags were extracted from a total of 403 full-length *var* genes that were sequenced from seven laboratory isolates in a study that explored sequence diversity and classification of PfEMP1 sequences ( Rask *et al.*, 2010). The dataset comprised sequences from 3D7, IT4, HB3, DD2 from Indochina, RAJ116 and IGH-CR14 from India, and the Ghanaian isolate PFCLIN. The sequence tags from these genes were classified based on the Cys/PoLV approach ( Bull *et al.*, 2007) and the block sharing group approach ( Bull *et al.*, 2008), and information on the upstream promoter region and DCs was derived from ( Rask *et al.*, 2010).

*Var2CSA* and sequences without 5’ upstream promoter regions classification (ups) information were removed, leaving 313 sequences.

### Mapping of *var* genes onto a network of shared polymorphic sequence blocks

A total of 1,548 published DBL *α* sequences was obtained from Kilifi ( Bull *et al.*, 2008, n=1226) and from published parasite genomes ( Rask *et al.*, 2010, n=313), together with three DC8 sequences from a study conducted in Tanzania ( Lavstsen *et al.*, 2012) and six sequences from “sig2” sequences from ( Bull *et al.*, 2005b). Sequences that shared 10 amino acid blocks were identified and used to draw a network of shared common sequences herein referred to as a block-sharing network. The block-sharing networks were generated using a described method ( Bull *et al.*, 2008) and were visualized using Pajek 5.01 ( Batagelj & Mrvar, 2004). A Perl script ( Supplementary File 2) was used to build the sequence networks. For the network of 1,548 tag sequences, *var* tag sequences in fasta format (Dataset: 1548_tags.fa; Githinji, 2017) was used as the input and the output file saved with a .net extension for import into Pajek. The Pajek project used for network analysis is included as Supplementary File 3.

### Definition of block sharing groups

The block sharing group (BS) classification of DBLα tags came from a sequence network analysis approach that aimed to visualize how different sequences share blocks of polymorphic sequence. Analysis of fully connected components of a sequence network constructed from observing the sharing of 14 amino acid blocks within DBLα tag sequences from parasites from Kenyan children showed that the largest component, called “block sharing group 1” (BS1) contained predominantly known upsA *var* genes. The second largest component was called block sharing group 2 (BS2) ( Bull *et al.*, 2008). We subsequently allocated the newly sequenced DBLα tags to BS1 or BS2 if they contained one or more sequence blocks from the originally defined block sharing groups 1 or 2. We further defined sequences with two cysteines that were classified as BS1 (cys2BS1) as “group A like” ( Warimwe *et al.*, 2009) and found that their expression was associated with cerebral malaria ( Warimwe *et al.*, 2012).

### Functional predictions from DBLα tag information

Receiver operator curves (ROC) were used to visualise the sensitivity and specificity of using specific subsets of DBLα sequence tags in the prediction of upsA, DC8, DC13 and CIDR1α, as outlined in Supplementary File 4.

The block sharing groups were originally defined using a global collection of sequences that included sequences from 3D7 and IT4 laboratory isolates ( Bull *et al.*, 2008); therefore, sequences from 3D7 and IT4 isolates were excluded in the block-sharing group analysis presented here. Statistical analysis was done using R version 3.4.0 as outlined in Supplementary File 1.

## Results and discussion

**Figure 1.**
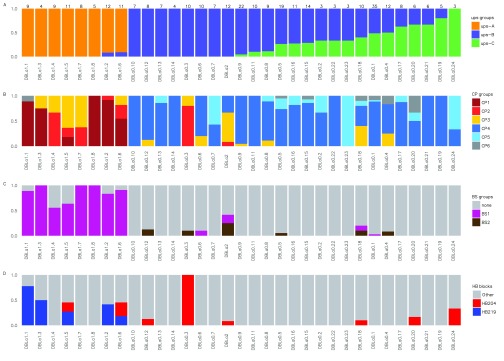
Correspondence between various *var* sequence classifications and possession of specific DBLα domains classified by ( Rask *et al.*, 2010), for *var* genes sequenced from 6 laboratory isolates. Each *var* gene contains only one DBLα domain. For each subset of *var* genes, classified according to their DBLα domains (x axis), the proportion of genes carrying other sequence features is shown (y axis). ( **A**) ups classification; ( **B**) cys/polv classification ( Bull *et al.*, 2005b); ( **C**) block sharing group classification ( Bull *et al.*, 2008); ( **D**) selected homology block classifications ( Rorick *et al.*, 2013). The domains are arranged from left to right in order of decreasing proportion of upsA to upsC-containing *var* gene sequences. The total number of sequences from each domain is shown at the top of the figure.

**Figure 2.**
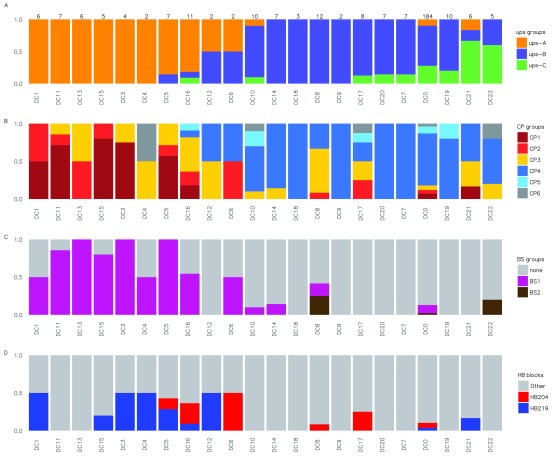
Correspondence between various *var* sequence classifications and possession of specific domain cassettes (DCs) for *var* genes sequenced from 6 laboratory isolates ( Rask *et al.*, 2010). For each subset of *var* genes, classified according to their DC (x axis), the proportion of genes carrying other sequence features is shown (y axis). ( **A**) ups classification; ( **B**) cys/polv classification ( Bull *et al.*, 2005b); ( **C**) block sharing group classification ( Bull *et al.*, 2008); ( **D**) selected homology block classifications ( Rorick *et al.*, 2013). The cassettes sorted from left to right such that the leftmost sequences contain the largest proportion of upsA *var* genes, while sequences to the right contain the largest proportion of upsC *var* genes. The number of sequences from each DC is shown at the top of the figure. Sequences that were not assigned to a domain are denoted as DC0.

**Figure 3.**
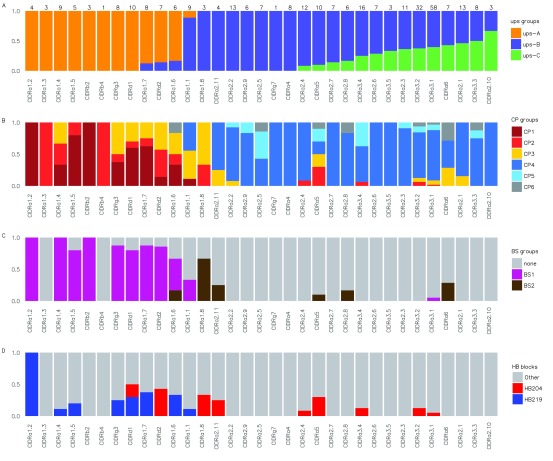
Correspondence between various *var* sequence classifications and possession of specific CIDR1 domains for *var* genes sequenced from 6 laboratory isolates ( Rask *et al.*, 2010). For each subset of *var* genes, classified according to their CIDR1 domains (x-axis), the proportion of genes carrying other sequence features is shown (y-axis). ( **A**) ups classification; ( **B**) cys/polv classification ( Bull *et al.*, 2005b); ( **C**) block sharing group classification ( Bull *et al.*, 2008); ( **d**) selected homology block classifications ( Rorick *et al.*, 2013). The CIDR domains are sorted from left to right, such that the left-most sequences contain the largest proportion of upsA, while sequences to the right contain the largest proportion of upsC *var* genes. The total number of *var* genes containing each of the CIDR1 domains is shown at the top of the figure.

**Figure 4.**
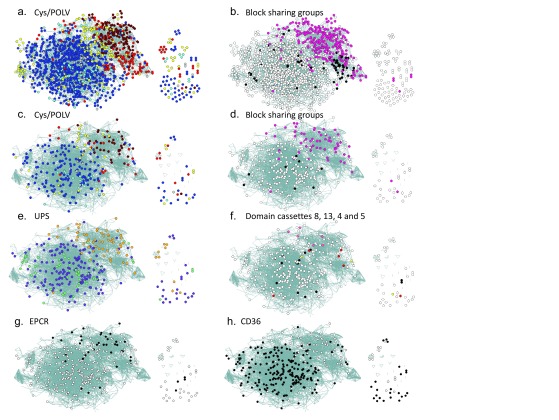
Network analysis of DBLα tag sequences collected from Kilifi ( Bull *et al.*, 2008), 6 laboratory isolates ( Rask *et al.*, 2010) and Tanzanian ( Lavstsen *et al.*, 2012). The analysis builds on that described in ( Bull *et al.*, 2008). ( **a**) Cys/polv analysis for all sequences; ( **b**) block sharing groups analysis for all sequences; ( **c**) Cys/polv analysis for full length *var* gene sequences from 6 laboratory isolates; ( **d**) block sharing groups analysis for full length *var* gene sequences from 6 laboratory isolates; ( **e**) ups grouping for full length *var* gene sequences from 6 laboratory isolates; ( **f**) domain cassette (DC) classification for DC4, DC5, DC8 and DC13 for full length *var* gene sequences from 6 laboratory isolates; ( **g**) predicted EPCR-binding phenotype due to CIDRα1.1, CIDRα1.4, CIDRα1.5, CIDRα1.6, CIDRα1.7 or CIDRα1.8 ( Lau *et al.*, 2015) for sequences with CIDRα information available; ( **h**) predicted CD36-binding phenotype due to CIDRα2, CIDRα3, CIDRα4, CIDRα5 ( Robinson *et al.*, 2003) for sequences with CIDRα information available. Colours of vertices match those defined in Figure 1: **a** and **c**) brown = cys/polv group 1 (CP1), red= CP2, yellow = CP3, blue = CP4, light-blue = CP5, grey = CP6; **b** and **d**) pink = block sharing group 1 (BS1), black = BS2, white = not a member of a block sharing group; **e**) orange = upsA, purple = upsB, light green = upsC; **f**) black = domain cassette 8 (DC8), red = DC5, pink = DC13, yellow = DC4; **g**) black = predicted EPCR binding; **h**) black = predicted CD36 binding.

**Figure 5.**
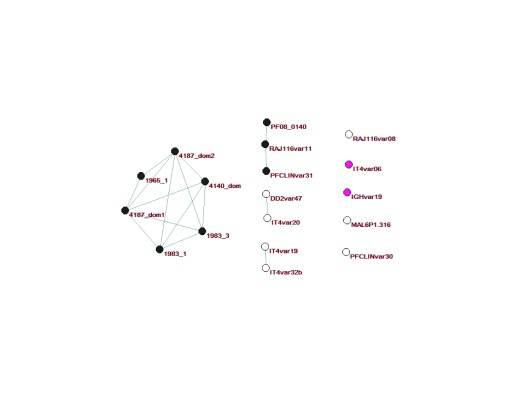
Network analysis of DBLα tag sequences from known DC8 *var* genes. sequences are from 6 genomes, DC8 Sequences 1983_3, 1983_1 and 1965_1 from a study in Tanzania ( Lavstsen *et al.*, 2012) and “sig-2” sequences from Kenya, 4140_dom 4187_dom1 and 4187_dom2 ( Bull *et al.*, 2005b). Colours of vertices match those defined in Figure 1: pink = block sharing group 1 (BS1); black = BS2; white = not a member of a BS.

**Figure 6.**
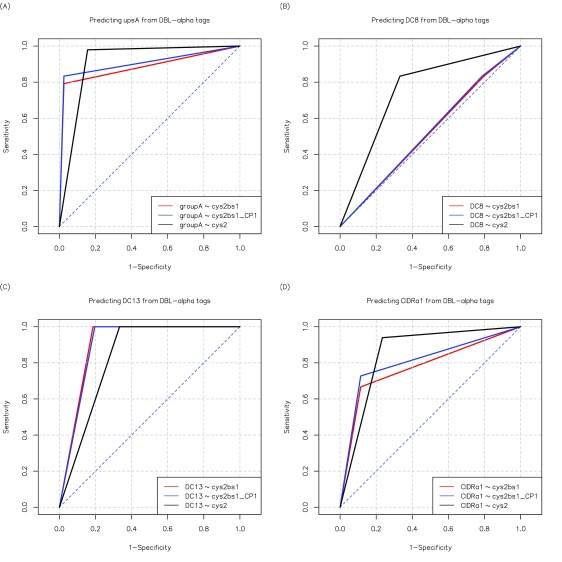
Receiver operator curves showing the sensitivity and specificity of three DBLα tag classifications in predicting *var* gene features associated with disease severity. ( **A**) Sensitivity and specificity in predicting upsA sequences. ( **B**, **C**) The prediction of DC8 and DC13 sequences. ( **D**) The prediction of CIDR1α domains from tag information. Sequences from 3D7 and IT4 were excluded from the analysis because they were used for developing these these classifications ( Bull *et al.*, 2008). cys2 = two cysteines within the tag region; cys2bs1 = tag sequences in block sharing group1 AND have two cysteines, defined as “group A-like” ( Warimwe *et al.*, 2009); cys2bs1_CP1 = cys2bs1 OR in cys/PoLV group 1.

Our aim was to summarize the relationships between sequence features within DBLα tag sequences, and sequence features available from fully sequenced *var,* genes from seven fully sequenced genomes ( Rask *et al.*, 2010). The relationships between these two levels of information were visualized using bar graphs ( Figure 1, Figure 2 and Figure 3; Figure S1 and Figure S2) a network visualization approach ( Figure 4 and Figure 5) and through a sensitivity, specificity analysis ( Figure 6).

Figure 1 focuses on 313 DBL domains classified by ( Rask *et al.*, 2010) into 33 DBLα sub-groups. The DBLα tag region within were classified by both the block-sharing ( Bull *et al.*, 2008) and the cys/polv ( Bull *et al.*, 2005b) classifications. The ups region of each corresponding gene is also shown. BS1 sequences were closely associated with upsA, and BS2 sequences were associated largely with upsB or upsC. While most cys2 sequences (CP1-3) were found within sequences containing the upsA promoter, some of them were also found in sequences containing upsB and upsC promoters. For example, sequences with DBLα-0.3 or DBLα-2 subdomains were largely upsB. However, they contained relatively high proportions of *var* sequences with two cysteines, specifically those from CP2 and CP3 Cys/PoLV groups.

### DBLα *sub-*domains are not all homogeneous groups

Domain classification that was suggested by ( Rask *et al.*, 2010) were partly based on global sequence alignments. Applying sequence alignment to a large collection of recombining *var* sequences is challenging because the alignment process does not consider the recombination history and potentially defines sequences as distinct when they are part of a network of recombining sequences.

Examination of DBLα tags suggests that MFK and REY motifs (highly enriched within subsequently defined homology blocks 219 and 204 ( Rask *et al.*, 2010; Rorick *et al.*, 2013)) are never found on the same sequence ( Bull *et al.*, 2005b). However, DBLα1.5, DBLα1.2 and DBLα1.6 groups defined by Rask and colleagues each comprise a mixture of MFK-containing and REY-containing sequences ( Figure 1). The domain classification used in ( Rask *et al.*, 2010) has therefore brought together distinct sequences within the same sequence classification. This suggests that the newly defined sub-domains do not always classify sequences into wholly genetically distinct groups. This discordance between methods of classification, employing global and local sequence comparisons reflects a mode of diversification of *var* sequences by *P. falciparum* that we might speculate leads to impaired recognition and clearance of PfEMP1 antigens by the immune system.

### Existing DBLα tag classification cannot predict DC8 sequences from a global sequence collection

Similar to group A-like sequences, DC8 sequences are associated with severe malaria ( Bengtsson *et al.*, 2013; Bertin *et al.*, 2013; Lavstsen *et al.*, 2012; Rask *et al.*, 2010) and contain a specific class of DBL *α*2 sequences that appear to result from recombination events at a recombination hotspot proposed to be situated 3’ of the DBLα tag region ( Lavstsen *et al.*, 2012). Low levels of linkage disequilibrium between the DBLα tag region and parts of the genes encoding important cytoadhesive regions potentially limits the predictive information available within DBLα tag sequence. This is consistent with the observation that DC8 sequences contain multiple cys/PoLV groups CP2, CP3 and CP4 ( Figure 2). However, none of the identified DC8 sequences contain CP1 tags, perhaps suggesting some level of linkage disequilibrium with the tag region. In support of this possibility, DC8 sequences contained the highest proportion of observed BS2 sequences of any DC. Furthermore, an additional set of DC8-like sequences identified in Tanzania ( Lavstsen *et al.*, 2012) were similar to previously defined “sig2” sequences found in two severe malaria cases sampled from Kenyan children ( Bull *et al.*, 2005b). Both sets of sequences are defined as BS2, CP2. We have previously suggested that BS2 sequences may be characteristic of *var* genes sampled from Africa ( Bull *et al.*, 2008). It is possible that DC8 sequences sampled from limited geographical regions may show significant levels of linkage disequilibrium with DBLα tag sequence features (see Figure 5 below).

### Mapping tag regions from full length *var* genes onto a network of DBLα tag sequences from Kenyan children

Patterns of diversification in sequences may give an indication of how these sequences evolve in the face of *in vivo* selection pressure. In Figure 4 and Figure 5, we used our previously described approach of visualizing the sharing of polymorphic blocks within DBLα to explore specific subsets of full length *var* genes.

To understand how various sequences with known DCs mapped to this network, we re-drew the network from ( Bull *et al.*, 2008) whilst including the sequences from the 7 genomes. We also supplemented the figure with additional sequences including, the “sig 2” sequences identified in a previous analysis of isolates causing severe and non-severe malaria and DC8 sequences identified in Tanzania ( Lavstsen *et al.*, 2012). As shown in Figure 4F, DC8 sequences were restricted mainly to the region of the network containing mainly upsB and upsC sequences, while DC13 were associated with the region of the network enriched in upsA sequences. Figure 5 further illustrates the relationships between DBLα tags from known DC8 genes.

Sequences with DC4 cassettes are reported to be associated with binding to ICAM1 ( Bengtsson *et al.*, 2013). In this data set, there were only 2 sequences with DC4 cassettes; one sequence has a CP3 DBLα tag region and the other a CP6 DBLα tag region ( Figure 4F). These sequences map to distinct locations within the network. Sequences with DC5 cassettes were from different Cys/PoLV groups all of which belonged to BS1, three of which mapped to a similar region of the network ( Figure 4F).

To map predicted cytoadhesive properties of the PfEMP1 antigens encoded by these genes, we made predictions based on existing information and mapped these cytoadhesive properties onto the network ( Figure 4). Endothelial protein C receptor binding and CD36 binding were predicted based on the binding properties of recombinant CIDR domains from ( Lau *et al.*, 2015) and ( Robinson *et al.*, 2003) respectively ( Figures 4G and H). Though the number of sequences is very limited, this mapping of predicted cytoadhesive properties is consistent with the idea that functional specialization of *var* genes is associated with broad sequence differences that are detectable within DBLα tag sequences.

A recent study ( Rorick *et al.*, 2013) has further explored this possibility by classifying DBLα tags using homology blocks defined in ( Rask *et al.*, 2010). They found in datasets from Kenya and Mali that homology block 204 (closely related to CP2) was associated with impaired consciousness and homology block 219 (closely related to CP1) was associated with rosetting. Figure 1 and Figure 2 also summarizes how these two homology blocks relate to other DBLα tag classifications.

### Sensitivity and specificity analysis

In summary, this analysis shows that some information about functionally relevant *var* gene sequence features from existing DBLα tag sequence classification methods. Most notably, the presence of a CIDRα1 domain, predicted to bind to endothelial protein C receptor ( Lau *et al.*, 2015) and associated with severe malaria ( Jespersen *et al.*, 2016) is associated with “group A-like” sequences (bs1cys2), which potentially explains previously reported associations between both the expression of related subsets of cys2 sequence tags and DC8 and DC13 *var* genes, with severe malaria ( Kirchgatter & Portillo, 2002; Kyriacou *et al.*, 2006; Warimwe *et al.*, 2012). Figure 6 summarizes sensitivity and specificity analyses for the associations described. Supplementary File 5 (Tables 1–12) shows the corresponding statistical significance. Figure 6 also illustrates the slightly increased sensitivity of prediction of presence of a CIDRα1 domain through expanding the definition of group A-like to include all CP1 sequences (cys2bs1_CP1). Associations between DBLα tag classifications and full length *var,* sequences are useful for bringing together and explaining findings from previous studies. However, such analyses will soon be replaced by methods such as RNAseq ( Otto *et al.*, 2010) or mass spectrometry ( Bertin *et al.*, 2013) that allow access to information from full length *var* genes and PfEMP1 sequences from clinical isolates.

## Data availability

The data referenced by this article are under copyright with the following copyright statement: Copyright: © 2017 Githinji G and Bull PC

The data and analysis scripts used in this analysis are available from OSF: http://doi.org/10.17605/OSF.IO/UWCN2 ( Githinji, 2017).

## Supplementary material

**Supplementary File 1:** R script - *var* expression analysis.

**Supplementary File 2:** Perl script - network constructor.

**Supplementary File 3:** Pajek project – network of 1548 *var* tag sequences.

**Supplementary File 4:** R script – sensitivity specificity analysis.

**Supplementary File 5:** Tables 1–12 show Fisher’s exact tests.

**Figure S1: Bar plots showing the distribution and proportion of block sharing groups (BS) across 23 domain cassettes.** All the full-length sequences that contained DC13 cassette architectures contained DBLα sequence tags that belonged to BS1. Full-length sequences with DC8 cassette architecture contained DBL sequence tags that belonged to either BS1 or BS2, and several the DBL tags did not belong either BS1 or BS2.

**Figure S2: The relationship between HB204, HB219 and cys/PoLV groups.**
