# Supplementary material for: A re-assessment of gene-tag classification approaches for describing var gene expression patterns during human Plasmodium falciparum malaria parasite infections
Source: Wellcome Open Res. 2017 Sep 19;2:86. [Version 1] doi: 10.12688/wellcomeopenres.12053.1 (PMC5635463; doi:10.12688/wellcomeopenres.12053.1)
Supplement: Supplementary file 5 [file wellcomeopenres-2-13039-s0004.tgz › b5e555f9-218e-4afd-8cc6-da7dcaaf4bac.pdf]

Supplementary File 5. Fisher's exact tests

	estimate	p.value	conf.low	conf.high	method	alternative
1	238.47	0.00	72.14	1028.22	Fisher's Exact Test for Count Data	two.sided

Table 1: Fishers exact test for cys2bs1 predicting upsA

	estimate	p.value	conf.low	conf.high	method	alternative
1	295.43	0.00	86.89	1329.73	Fisher's Exact Test for Count Data	two.sided

Table 2: Fisher's exact test for cys2bs1_CP1 predicting upsA

	estimate	p.value	conf.low	conf.high	method	alternative
1	399.56	0.00	63.87	14586.53	Fisher's Exact Test for Count Data	two.sided

Table 3: Fisher's exact test for cys2 predicting upsA

	estimate	p.value	conf.low	conf.high	method	alternative
1	0.42	0.70	0.01	2.99	Fisher's Exact Test for Count Data	two.sided

Table 4: Fisher's exact test for cys2bs1 predicting DC8

	estimate	p.value	conf.low	conf.high	method	alternative
1	0.40	0.70	0.01	2.85	Fisher's Exact Test for Count Data	two.sided

Table 5: Fisher's exact test for cys2bs1_CP1 predicting DC8

	estimate	p.value	conf.low	conf.high	method	alternative
1	4.94	0.01	1.28	23.00	Fisher's Exact Test for Count Data	two.sided

Table 6: Fisher's exact test for cys2 predicting DC8

	estimate	p.value	conf.low	conf.high	method	alternative
1	Inf	0.00	5.94	Inf	Fisher's Exact Test for Count Data	two.sided

Table 7: Fisher's exact test for cys2bs1 predicting DC13

	estimate	p.value	conf.low	conf.high	method	alternative
1	Inf	0.00	5.67	Inf	Fisher's Exact Test for Count Data	two.sided

Table 8: Fisher's exact test for cys2bs1_CP1 predicting DC13

	estimate	p.value	conf.low	conf.high	method	alternative
1	Inf	0.00	2.83	Inf	Fisher's Exact Test for Count Data	two.sided

Table 9: Fisher's exact test for cys2 predicting DC13

	estimate	p.value	conf.low	conf.high	method	alternative
1	14.98	0.00	6.98	33.24	Fisher's Exact Test for Count Data	two.sided

Table 10: Fisher's exact test for cys2bs1 predicting CIDRa1

	estimate	p.value	conf.low	conf.high	method	alternative
1	17.99	0.00	8.29	40.64	Fisher's Exact Test for Count Data	two.sided

Table 11: Fisher's exact test for cys2bs1_CP1 predicting CIDRa1

	estimate	p.value	conf.low	conf.high	method	alternative
1	34.05	0.00	12.64	115.93	Fisher's Exact Test for Count Data	two.sided

Table 12: Fisher's exact test for cys2 predicting CIDRa1
