# Supplementary figures and images for: A re-assessment of gene-tag classification approaches for describing var gene expression patterns during human Plasmodium falciparum malaria parasite infections
Source: Wellcome Open Res. 2017 Sep 19;2:86. [Version 1] doi: 10.12688/wellcomeopenres.12053.1 (PMC5635463; doi:10.12688/wellcomeopenres.12053.1)

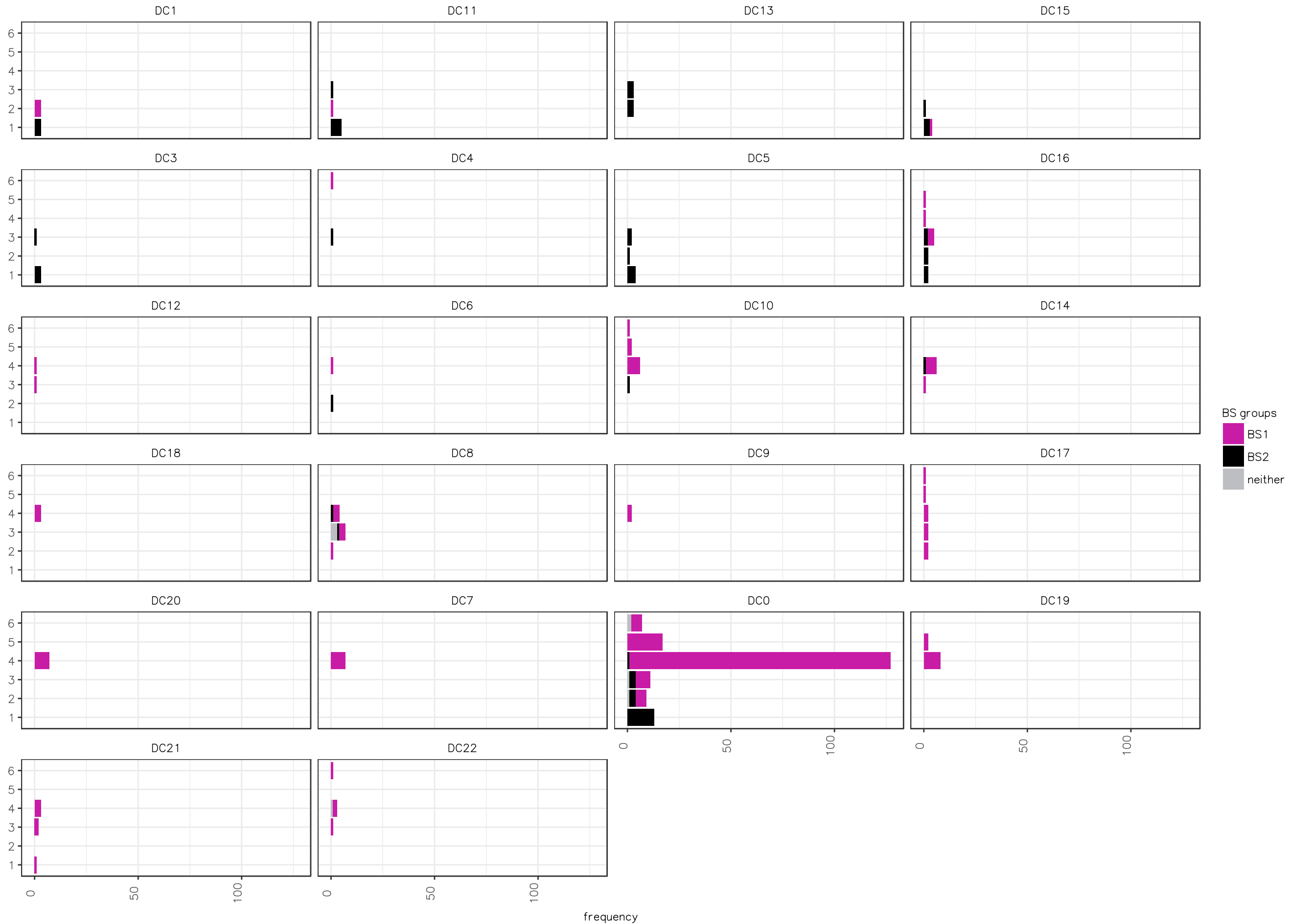

Supplement: Supplementary file 6 [file wellcomeopenres-2-13039-s0005.tgz › b1c36d79-4c27-4b72-8604-6324a84fa33a.pdf]

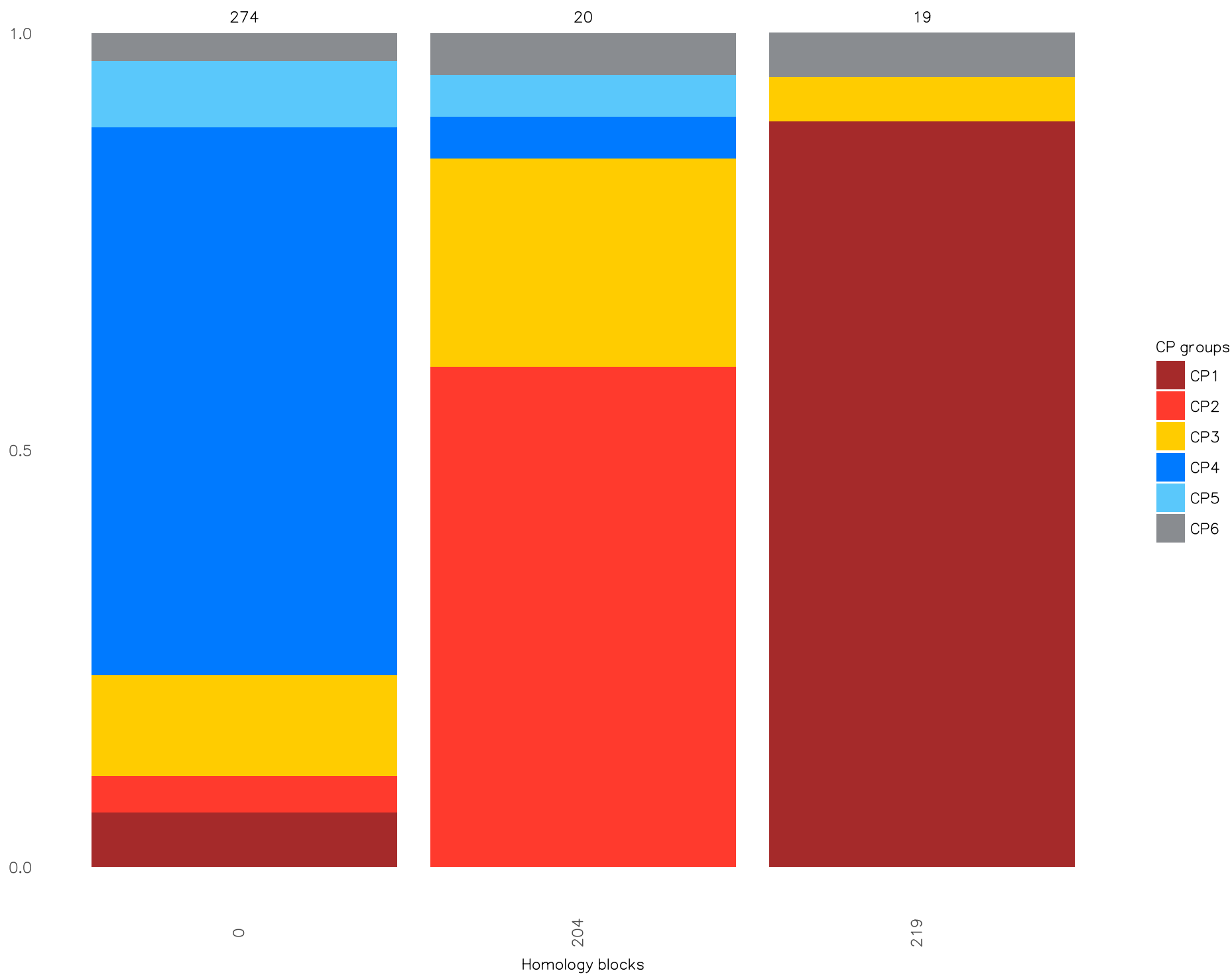

Supplement: Supplementary file 7 [file wellcomeopenres-2-13039-s0006.tgz › 5f54d62b-e0ad-4f21-a6ae-b0f4ce4282f1.pdf]
