# Supplementary material for: A re-assessment of gene-tag classification approaches for describing var gene expression patterns during human Plasmodium falciparum malaria parasite infections
Source: Wellcome Open Res. 2017 Sep 19;2:86. [Version 1] doi: 10.12688/wellcomeopenres.12053.1 (PMC5635463; doi:10.12688/wellcomeopenres.12053.1)
Supplement: Peer review history

# Peer review: Referee response for version 1

**DOI:** 10.21956/wellcomeopenres.13039.r26166

**Published:** 2017-10-09

**Authors:** Thomas Lavstsen

**Affiliations:** 1 Centre for Medical Parasitology, Department of Immunology and Microbiology (ISIM), Faculty of Health and Medical Sciences, University of Copenhagen, Copenhagen, Denmark

Githinji and Bull present an analysis of the associations between previously developed annotation tools whole PfEMP1 sequences and for the “DBLa-tag”, a short PCR-amplifiable sequence found in all PfEMP1 encoding genes allowing unique identification of specific var genes. Reconciling sequence traits of known PfEMP1 receptor binding phenotype, defined PfEMP1 domain types and DBL-tag annotation methods is important as despite advances in high throughput sequencing, analysis of DBLa-tag along with qPCR analysis still represent most efficient and precise detection of the polymorphic var genes expressed by parasites in patients.

 The paper presents the analyses in a set of intuitively easily understood graphs, - however PfMEP1 domain composition and nomenclature can easily become confusing. Most of my comments and suggestions relate to improvement and corrections of explanations towards a simpler and hopefully clearer presentation of the current knowledge and relevance of this study. One additional analysis regarding adding predicted ICAM1 binding PfEMP1 should be added to one of the figures.

 Specific comments:

 Although shown many times, it will be useful to have a very simple diagram showing PfEMP1 domain structure indicating the position of the DBLa-tag, the known hotspots of recombination at the DBLa-tag end, and mid var region. This will highlight the purpose and challenge of this whole exercise.

 Quote: “3) Analysis is further complicated by the high diversity of each domain subclass and lack of clear associations between specific adhesion phenotypes and classes of domains.”

 I would phrase this differently. Although many binding phenotypes have been proposed for iRBCs, well characterized interactions for PfEMP1 are more limited. In fact only few interactions are studied to the extent that these can be used to predict PfEMP1 function: CSA, CD36, EPCR and ICAM1. For these there is only a small uncertainly for determining ICAM1 binding domains.

 Binding to HABP1, PECAM1, IgM, etc is, as stated not clearly linked to specific domains or sequence traits. As parasite adhesion phenotypes cannot be investigated in vivo, "specific adhesion phenotypes" is defined from a combination of clear association of PfMEP1 domain type with binding to a specific receptor, as well as validation of this by iRBC binding assays; and thus observed iRBC binding to various receptors cannot be taken as a gold standard on its own.

 I suggest Correcting next paragraph to: Based on full-length sequences from seven laboratory isolates, each domain class was divided through global sequence alignment into further sub-classes (Rask *et al., *2010). For example, the DBLa domains were reclassified into 33 sub-domains (DBLa0.1 - 0.24, DBLa1.1 - 1.8 and DBLa2).

 I suggest changing references as: “ *Ups *E is associated exclusively with *var2CSA*(Lavstsen *et al., *2003), which plays a central role in placental malaria (Reference Salanti et al J ex Med).

 Quote: “It was initially proposed that DCs may act as functional units. However, clearly defined functions have only been assigned at the level of individual domain sub-classes. Therefore, though common combinations of domains exist, it is unclear whether they represent functional units.”

 I agree with this. I would even suggest that it is clear that most domain cassettes, although useful to define molecular tools, does not appear to reflect functional units. However, I do not think the examples given does not elude clearly to this. In line with my comment above, I think it should be made clear that the binding phenotypes we understand well today, (EPCR; CD36 and ICAM1) all are associated with and fully contained within single domains. However, some subsets of these domains appear to have co-evolved - like ICAM1 binding (DBLb) in group A is always found in CIDRa1 (EPCR binding) variants, (co-evolution clearly seen within the DC13 context); whereas ICAM1-binding DBLb are rarely found in CIDRa1 (EPCR binding) containing DC8; and DBLb are not specifically associated with any Domain subclass when found in group B.  The comment of DBLb domains being targets of cross reactive antibodies seems irrelevant in this context and confusing here.

 Paragraph. “Clinical and laboratory studies have reported…..”.

 The DC8 and DC13 are useful for understanding how these variable genes can be probed and detected iin vivo. But given the limited usefulness of the DCs to describe known binding phenotypes, and their relation clinical outcome;

 And the recent studies of var expression (Those referenced, and the Mkumbaye et al IAI 2017, not included but should be) - which are in line with previous work as described in two paragraphs before;

 And to simplify for those new to the field –

 I think it would be best to use this paragraph to describe the consensus from these studies, that CIDRa1 is the only common trait of var genes whose expression is associated with malaria pathology; regardless of symptomology. And EPCR+IMCA1 but not CD36+icam1 is found more frequently in CM (lennartz et al 2017). These findings does not mean that future studies relying on DBLa tag is not useful or needed.

 M&M section: “Definition of block sharing groups”

 This is a more detailed (and required) description of the statement above on SB1, correct? Perhaps just refer back to this. It is unclear which part of the text refers to previous work or and which to the re-analysis performed here. Leave out the sentence: “and found that their expression was associated with cerebral malaria (Warimwe *et al., *2012).”. This is not relevant here.

 I think following concluding statement is not needed ,as the whole premise for the study is to compare classification methods,  - it is given or should already be stated that the sequences has evolved to under pressure to diversify in response to immune recognition, and to maintain structural fold to retain function: “This discordance between methods of classification, employing global and local sequence comparisons reflects a mode of diversification of *var *sequences by *P. falciparum *that we might speculate leads to impaired recognition and clearance of PfEMP1 antigens by the immune system.

 “Similar to group A-like sequences, DC8 sequences are associated with severe malaria (Bengtsson *et al., *2013; Bertin *et al., *2013; Lavstsen *et al., *2012; Rask *et al., *2010)……”

 The main point to iterate here should be that DC8s have CIDRa1 and a recombined B/A DBLa domain named DBLa2. The link to SM is part of this fact; . ie the presence of the CIDRa1 domain.

 I am not sure which part the Bengtsson et al 2013 reference plays here.

 Also, specify that DC8 is (the only known) a B/A recombination  - thus B from UPS to DBLa tag end, and A like in its DBLaS3 and downstream from there. This is important to understand, as the A vs B/C grouping is tied to the chromosomal localization of these genes which ensures that A does not normally recombine with B (otherwise lethal chromosomes will be formed). This is probably why the DC8 is the best conserved domain cassette, and why DBLa tag analysis is particularly difficult (and important) to apply for these genes.

 “Sequences with DC4 cassettes are reported to be associated with…..”.

 The ICAM1 prediction has been refined considerable in Lennartz et al 2017. The authors should color in sequences which contain group B DBLb5s show/predicted to bind ICAM1 and the DBLb3/1 domaains found and predicted to bind ICAM1 (Lennartz et al; + the IT4var07 shown in PMID: 26119044 & PMID: 27406562 ; which I believe was not included in Lennarz et al).

  “Sequences with DC5 cassettes were from different…”

 ..As expected from this C terminal group A domain cassette - also previously described as not associated with N terminal seq features (Rask et al 2010).

 “However, such analyses will soon be replaced by methods such …”

 I disagree with this prediction. RNAseq may indeed prove useful if costs are reduced further to allow enough depth perform the required assembly of var genes, which are few and rare in RNA exptracted from blood in vivo; RNA seq may prove useful if the upcoming analysis of Sangers 1000 Pf genomes suggest so. Using MS analysis it is extraordinarily difficult to do de novo assembly of multiple rare polymorphic sequences in a patient sample, which is what is required to elude further to current knowledge. On the contrary I think this work lays the ground for a developing a much more cost effective var type prediction tool using DBLa tag expression analysis, once such a tool could be developed and validated from the ~1000 pf genomes to be released from Sanger.

I have read this submission. I believe that I have an appropriate level of expertise to confirm that it is of an acceptable scientific standard.

# Peer review: Referee response for version 1

**DOI:** 10.21956/wellcomeopenres.13039.r26465

**Published:** 2017-10-06

**Authors:** Mary M. Rorick

**Affiliations:** 1 Department of Ecology and Evolution, University of Chicago, Chicago, IL, USA

A nice computational study comparing multiple methods of categorization for the ultra-diverse, biologically complex, and clinically important family of *var *genes of the malaria parasite *P. falciparum*. The *var *genes encode *Plasmodium falciparum* Erythrocyte Membrane Protein 1 ( *Pf*EMP1). Due to the diverse, recombining nature of the *var*, from non-laboratory stains it is typically only feasible to sample a very short tag region of about 125 amino acids from the relatively conserved *var *DBLα domain. Therefore, most of the *var/Pf*EMP1 sequence variation currently available from the clinical setting consists of only this tag region. This region does not include most of the variable host endothelial binding sites that have been proposed in the literature, and which are potentially relevant to severe malaria disease. The careful mapping of the relationships between different *Pf*EMP1 classification schemes is therefore important for deciphering this protein’s multiple and variable binding functions, and the various disease manifestations that likely result as a consequence. Until it is possible to obtain the complete *var*/ *Pf*EMP1 sequences from large numbers of clinical isolates, this type of study is of useful for the progression of the field.

 The research methods appear to be of high quality, and the paper is clear and well-written. However, there are some typos and areas where the writing could be improved. Due to the complex nature of the topic I have included many detailed suggested edits below. The majority of the suggested changes are simply to improve the clarity of the manuscript - something that is important for readers from outside the community of scientists who study *var*/ *Pf*EMP1. 

1. This paper relies heavily on the results of Rask et al. 2010, which lay an extensive groundwork for *var*/ *Pf*EMP1 categorization. In that paper, the authors discuss the association between HB36 and cys4/cp4-6 sequences. The authors could maybe include a discussion of HB36 when they talk about predicting *ups*A/CIDRa1.
2. Throughout the manuscript, I believe PfEMP1 should be formatted with Pf in italics: *Pf*EMP1.
3. The authors explain the diversity and mosaicism of *var *genes as follows:  “due to recombination between *var* genes on non-homologous chromosomes, the overall architecture of PfEMP1 encoded by different parasite genotypes is extremely diverse and sequences are mosaics of many semi-conserved sequence blocks”. I think this is slightly overstating our current knowledge of the genetic mechanisms and evolutionary and ecological dynamics shaping and maintaining var diversity. Why the variants have a mosaic nature is likely party due to ectopic recombination (recombination between *vars* on non-homologous chromosomes), but also likely due to recombination between *vars* at the *same* genetic location within *homologous *chromosomes. *Var* genes surely recombine in a homologous manner at least as frequently as they recombine ectopically, and due to the diversity among different parasites even at a single *var *locus, this more normal type of recombination is also likely to generate mosaicism. Another reason it is an overstatement/misstatement: if we were going to give a reason for why there is all the *var* diversity, balancing selection is a much more direct explanation as opposed to non-homologous recombination. Balancing selection must be invoked to explain why so much *var* diversity is maintained within the population. In my view at least, the question of the “extreme diversity” of the *var *genes is not really addressed at all by just invoking the immediate genetic mechanisms generating the variants. Given the above, I recommend simply removing the following clause from the sentence: “due to recombination between *var *genes on non-homologous chromosomes”, and replacing it with: “due to rapid recombination among *var* genes, and likely balancing selection” .
4. The authors use the phrase “the infecting parasite population” twice near the end of the second paragraph of the introduction, and in both cases I believe they specifically mean the parasites within an single, individual host. I think this phrasing is more confusing than it needs to be. For any system with population-level dynamics occurring within organisms there is the possibility for confusion about the level of hierarchy the dynamics are operating on. Specifically, in this case, I think some readers may think the authors mean the population of infective parasites rather than only those parasites that exist simultaneously within a given host individual. Another possible source of confusion is that some readers may be familiar with the fact that the *var* genes are expressed in a strictly mutually exclusive manner, and it may not be obvious that this does not translate to strict mutually exclusive expression at the level of the host individual (also I believe some early clinical results have contributed to some of this confusion). I suggest rephrasing as follows: “multiple *var *genes are expressed simultaneously within the infecting parasite population” could be changed to “while *var* genes are expressed in a strictly mutually exclusive manner at the level of the individual cell, multiple *var* genes are expressed simultaneously at the level of the infected host”; and “The range of *var *genes expressed at any one time in the infecting parasite population” could be changed to “The range of *var *genes expressed at any one time within a given host”.
5. Starting with the third paragraph of the introduction I felt the structure of the manuscript begins to get a bit confusing. I had to work too hard as a reader to follow where they were going with the introduction, and why they were presenting this information in this order. I therefore suggest simplifying the writing in the third, fourth and fifth paragraphs of the introduction, and giving the reader a bit more of an explicit “road map” indicating how the paragraphs are connected and where they are taking us. Specific suggestions follow.
6. A topic sentence could be added to the very beginning of the third paragraph: “The hope has been that it may be possible to identify a limited set of *Pf*EMP1 functional specializations, which may in turn clarify the disease process; however, it remains unclear which aspects of *var *diversity are the most relevant for achieving this goal.”
7. For simplicity, I suggest deleting “Based on full length sequences from seven laboratory isolates …”, adding the topic sentence suggested above, and changing the original first sentence of the paragraph (which would now be the second sentence of the paragraph) to the following: “All *Pf*EMP1 domain classes have been classified through global sequence alignment into a large number of highly refined and specific domain sub-classes (Rask et al., 2010).”
8. The second sentence of the third paragraph of the Introduction is not a grammatically well-formed sentence. Also it mentions 33 sub-domains, which implies regions smaller than a domain, but I believe that is not what the authors mean. I believe they mean domain sub-classes (i.e., smaller, more refined categories of domains). I suggest changing the first part of the sentence to the following: “For example, the DBLα domain can be classified into 33 domain sub-classes….”
9. I suggest rewriting the fourth paragraph as follows: “In addition to the refined domain classification schemes—which were based on the handful of sequenced laboratory strains for which we have complete *var *sequences—various *broader* classification methods have also been employed for the *var* genes that use a sparser set of their sequence features. *Pf*EMP1 genes can be classified into just five broad functional and recombination groups based on sequence similarity of their upstream promoter regions ( *ups*) and chromosomal location. This classification partitions the sequences into groups A-E (citations). *UpsE *is associated exclusively with *var2CSA*, which plays a central role in placental malaria (citation). *UpsA var* gene [note typo correction here] expression is associated with severe disease (citations) and rosetting (citations). Increased transcription of *upsB* sequences have also been reported in cases of severe malaria (citation). And, while some research indicates that *upsC* sequences are expressed at higher levels in asymptomatic cases (citations), it has also been reported that *upsC* expression is associated with severe malaria (citation).”
10. Choose a consistent notation for *ups* groups (i.e. with or without italics, and with or without a space).
11. Choose whether to say “subclasses/subclass” or “sub-classes/sub-class”. Both are used in the manuscript, and consistency is the important thing. I prefer the word without the hyphen, but its just personal preference.
12. Again, mostly just for clarity, I suggest rewriting the fifth paragraph of the introduction as indicated below. I removed the “for example” and the numbering because, while the information is relevant, it is not clear to me that they are really *examples* of the initial statement.
  “ *Pf*EMP1 can also be described in terms of common configurations of different subclasses of domains. These common configurations are called “domain cassettes” (DCs) (Rask et al, 2010). Twenty-three *var* DCs have been defined from full-length domain alignments of sequences from seven laboratory strains. It was initially proposed that DCs may act as functional units. However clearly defined functions have still only been assigned at the level of individual domain subclasses. Therefore, it remains unclear whether DCs represent functional units under natural selection, or whether they are just neutral artifacts of the recombinatoric diversification process. [Paragraph break here.] Research pertaining to DCs has revealed the following: Specific CIDRα1 domains, often found in the context of domain cassette 8 (DC8) and 13 (DC13), appear to bind EPCR (citation). Var genes containing DC8 cassettes seem to bind human endothelial cells from various organs, including—notably—those from brain endothelial cells (citations). DBLβ domains found within DC4 genes reportedly adhere to ICAM-1 and may be targets of broadly cross-reactive and adhesion-inhibitory IgG antibodies (citations). [Remove paragraph break that is currently here.] Clinical and laboratory studies have reported associations between DCs....”
13. Page 3, second column, fifth line from the bottom: I would remove the word “respectively”.
14. Page 4, first column, first paragraph: “by simply partitioning tags to those with and those without two cysteins” does not make grammatical sense, and it implies 2 versus 0 cysteins, which I believe is not what the authors mean. This could be changed to: “by simply partitioning tags by whether they contain two cysteins or some other number of cysteins”.
15. Page 4, first column, line 9: I would remove “Furthermore”.
16. Page 4, first column: For clarity, I recommend changing the sentence “Furthermore, Lavstsen et al., 2012 have suggested that information on EPCR binding by CIDRα1 within DC8 and DC13 may be unavailable within the DBLα tag due to a recombination hotspot situated between the DBLα tag region and the CIDRα domain” to the following: “Lavstsen et al., 2012 have suggested that the DBLα domain tag may not be informative about whether its flanking CIDRα1 domain binds EPCR because there is a recombination hotspot situated between the DBLα tag region and the CIDRα1 domain” [also, note that I changed CIDRα to CIDRα1 since I thought that was likely a typo].
17. When referring to a paper within a sentence (as opposed to the parenthetical manner at the end of a sentence) the authors sometimes use no parentheses: “Lavstsen et al., 2012 have suggested…” and other times use parentheses: “classification that was suggested by (Rask et al., 2010)”. The style should at least be consistent, and ideally also consistent with the journal’s formatting recommendations for this type of citation.
18. At least in two places there is a weird comma after “ *var”* that does not appear to belong: Within the first paragraph of “Results and discussion”, and within the 6 ^th^ line up from the very end of the manuscript.
19. Final line of the first paragraph of “Results and discussion”: I recommend changing “sensitivity, specificity analysis” to “sensitivity-specificity analysis”.
20. For consistency and clarity, use italics for “ups” or spell it out and don’t use the abbreviation. For example, page 4, second column, 7 ^th^ line up from the bottom.
21. I believe there is an error in the title of the section “DBLα sub-domains are not all homogeneous groups”. I believe it should read “DBLα domain subclasses are not all homogeneous groups”. A “sub-domain” and a domain “sub-class” are completely different things. The first is physically smaller than a domain, the second is a smaller category of a complete domain. As far as I can tell, everywhere the authors use the term “sub-domain” they actually mean “domain sub-class” (or equivalently “domain subclass”).
22. First sentence of the section “DBLα sub-domains are not all homogeneous groups”: Grammatical errors. Insert “The” before “Domain classification that was suggested…”, and replace “were” with “was”.
23. Page 5, second column: These two sentences feel pretty meaningless to me, plus it seems the meaning the authors are trying to convey is redundant with the sentences the precede and follow. Therefore I would just delete both of the sentences: “The domain classification used in (Rask et al., 2010) has therefore brought together distinct sequences within the same sequence classification. This suggests that the newly defined sub-domains do not always classify sequences into wholly genetically distinct groups.”
24. I think “This discordance between methods of classification, employing global and local sequence comparisons…” would read more clearly as follows: “This discordance between methods of classification when employing global versus local sequence comparisons…”
25. The above sentence continues by describing “a mode of diversification… that we might speculate leads to impaired recognition and clearance of PfEMP1 antigens by the immune system.” I think this sentence presents an interesting biological hypothesis that could be elaborated on more. To me it is not immediately obvious that the discordance should be interpreted in this biological manner. An alternative interpretation might be that some categorization methods are less informative about recombination patterns, or more noisy, for example. I simply find the hypothesis interesting and warranting of further discussion.
26. Page 6, first column: I recommend changing: “recombination hotspot proposed to be situated 3’ of the DBLα tag region” to “recombination hotspot purportedly situated 3’ of the DBLα tag region”.
27. Page 6, first column: I recommend changing: “DBLα tag region and parts of the genes encoding important cytoadhesive regions potentially limits the predictive information available with DBLα tag sequence” to “DBLα tag region and **the** parts of the **gene **encoding important cytoadhesive regions potentially limits the predictive information available with **the **DBLα tag sequence”.
28. Page 6, first column: I recommend adding a colon to “multiple cys/PoLV groups CP2, CP3 and CP4” so it reads “multiple cys/PoLV groups: CP2, CP3 and CP4”.

I have read this submission. I believe that I have an appropriate level of expertise to confirm that it is of an acceptable scientific standard.
